# Controlling Schistosomiasis: Significant Decrease of Anaemia Prevalence One Year after a Single Dose of Praziquantel in Nigerien Schoolchildren

**DOI:** 10.1371/journal.pntd.0000241

**Published:** 2008-05-28

**Authors:** Zilahatou B. Tohon, Halima B. Mainassara, Amadou Garba, Ali E. Mahamane, Elisa Bosqué-Oliva, Maman-Laminou Ibrahim, Jean-Bernard Duchemin, Suzanne Chanteau, Pascal Boisier

**Affiliations:** 1 CERMES/Réseau International des Instituts Pasteur, Niamey, Niger; 2 Programme National de Lutte contre la Bilharziose et les Géohelminthes, Niamey, Niger; 3 Schistosomiasis Control Initiative, Imperial College, London, United Kingdom; 4 Institut Pasteur, Nouméa, Nouvelle Calédonie; 5 Centre Pasteur du Cameroun, Yaoundé, Cameroon; Imperial College Faculty of Medicine, United Kingdom

## Abstract

**Background:**

In the framework of the monitoring and evaluation of the Nigerien schistosomiasis and soil-transmitted helminth control programme, a follow-up of children took place in eight sentinel sites. The objective of the study was to assess the evolution of *Schistosoma haematobium* infection and anaemia in schoolchildren after a single administration of praziquantel (PZQ) and albendazole.

**Methods/Principal Findings:**

Pre-treatment examination and follow-up at one year post-treatment of schoolchildren aged 7, 8, and 11 years, including interview, urine examination, ultrasound examination of the urinary tract, and measurement of haemoglobin. Before treatment, the overall prevalence of *S. heamatobium* infection was 75.4% of the 1,642 enrolled children, and 21.8% of children excreted more than 50 eggs/10 ml urine. Prevalence increased with age. The overall prevalence of anaemia (haemoglobin <11.5 g/dl) was 61.6%, decreasing significantly with increasing age. The mean haemoglobinemia was 11 g/dl. In bivariate analysis, anaemia was significantly more frequent in children infected with *S. haematobium*, although it was not correlated to the intensity of infection. Anaemia was also associated with micro-haematuria and to kidney distensions. In a sub-sample of 636 children tested for *P. falciparum* infection, anaemia was significantly more frequent in malaria-infected children. In multivariate analysis, significant predictors of anaemia were *P. falciparum* infection, kidney distension, and the village. One year after a single-dose praziquantel treatment (administered using the WHO PZQ dose pole) co-administered with albendazole (400 mg single dose) for de-worming, the prevalence of *S. haematobium* infection was 38%, while the prevalence of anaemia fell to 50.4%. The mean haemoglobinemia showed a statistically significant increase of 0.39 g/dl to reach 11.4 g/dl. Anaemia was no longer associated with *S. haematobium* or to *P. falciparum* infections, or to haematuria or ultrasound abnormalities of the urinary tract.

**Conclusions:**

The high prevalence of anaemia in Nigerien children is clearly a result of many factors and not of schistosomiasis alone. Nevertheless, treatment of schistosomiasis and de-worming were followed by a partial, but significant, reduction of anaemia in schoolchildren, not explainable by any other obvious intervention.

## Author Summary

The World Health Organization's recommendation for the control of urinary schistosomiasis is to reduce morbidity by reducing the prevalence of heavy infections. In Niger, where urinary schistosomiasis is endemic along the Niger River valley and in proximity to ponds, a national control programme for schistosomiasis and soil-transmitted helminth was launched in 2004 with the financial support of the Gates Foundation through the Schistosomiasis Control Initiative. In the framework of the monitoring and evaluation of the control programme, a follow-up of school children took place in eight sentinel sites. The aim of this study was to assess the evolution of *Schistosoma haematobium* infection and associated morbidity after a single-dose administration of praziquantel and albendazole. Before treatment, the overall prevalence of *S. heamatobium* infection was 75.4% and anaemia (haemoglobin <11.5 g/dl) was present in 61.6% of the study sample. One year after a single-dose praziquantel treatment (administered by dose-pole) co-administered with albendazole (400 mg single dose) for de-worming, all morbidity markers of the infection decreased significantly. This study shows how a schistosomiasis control programme can benefit populations by improving their health status.

## Introduction

*Schistosoma haematobium* is endemic in Niger, particularly throughout the Niger River valley and in villages in close proximity to permanent and temporary ponds [1]–[3]. The prevalence of infection is highly variable from one village to another. School-aged children are more often and more heavily infected than adults [4]. Overall, urinary schistosomiasis is an important public health problem in Niger. Thanks to portable ultrasound technology, urinary tract lesions due to *S. haematobium* infection can be easily identified in large epidemiological surveys [5]–[8]. Nevertheless, the relationship between other frequent pathologies, such as anaemia and *S. haematobium* infection, are less thoroughly documented [9]. Different studies have found a statistically significant association between urinary schistosomiasis and anaemia [10],[11]. However, many other factors, such as malaria, soil-transmitted helminthiasis, nutritional deficiencies and sickle-cell anaemia, are also frequently associated with anaemia [12]–[15] and controversial statements have been made about the real impact of schistosomiasis on haemoglobin status.

In the framework of the evaluation of the Nigerien national schistosomiasis and soil-transmitted control programme, launched in 2004 with the financial support of the Schistosomiasis Control Initiative (SCI), a longitudinal survey was implemented prior to the first round of mass treatment. This treatment consisted of the administration of praziquantel and albendazole to all the target population (school-aged children and high risk groups) determined by the programme. The double aim of this survey was to collect baseline data on parasitological and morbidity indicators and to monitor their evolution.

## Methods

### Population

Eight villages located in schistosomiasis endemic regions were randomly selected to represent the two main transmission patterns in Niger: six villages located near permanent (Tabalak, Kokorou) or semi-permanent (Kaou, Mozague, Rouafi, and Sabon Birni) ponds and two (Saga Fondo, Sanguile) located along the Niger River. The villages represented the south-western region (Tillabéry) and the central-northern region (Tahoua) of the country, with four villages from each region. One village is located in the Sudanian climatic zone and the seven others are in the Sahelian climatic zone.

Transmission is permanent along the river and near permanent pond settings while it is intermittent near temporary pond setting. The cold season (November to March) represents the high transmission period for both transmission patterns [16].

Both male and female schoolchildren aged 7, 8 and 11 years were the study population. These particular ages were selected to be included in the monitoring and evaluation surveys because the control programme supported by the SCI targets schoolchildren and therefore these age groups would be the most representative. The chosen study population would also have the lowest likelihood of high drop-out rates over the survey years, particularly those aged 7–8 years at baseline who will stay in primary school until they the age of 11–12 years old. Furthermore, if an improvement is evident in the study population than there is a higher probability that morbidity in adults can be prevented.

The recommended sample size was 60 children by age group in *S. haematobium* zones. When the sample size could not be attained with enrolled schoolchildren, further school-aged children were recruited from the village.

### Ethical considerations

The study was approved by the National Ethics Committee of Niger and the National Health System Local Research Ethics Committee of St. Mary's Hospital, London, prior to data collection. Once institutional consent obtained, meetings were held in all targeted villages in order to clearly present the objectives and the methods of the study to the families. They were invited to give their consent prior to inclusion of their children in the survey and they were made aware that they could refuse to permit their children from participating in the study or to remove their children without any consequences. The consent sought was verbal because of the high illiteracy rate in rural population, and the National Ethics Committee of Niger approved this method of consent.

Participating schoolchildren underwent a standardized interview focusing on clinical signs of urinary or intestinal schistosomiasis and on previous anti-schistosomal treatment.

### Urine and stool samples

From each child, a urine sample was collected between 10:00 AM and 2:00 PM and the presence of blood was noted, according to a standardized colorimetric scale coding from 0 to 6. Gross haematuria was defined as a visual quantification coded >3. Reagent strips (Hemastix Bayer Diagnostics Division, Tarrytown, NY, U.S.A) were used for diagnosing microscopic haematuria. Codes ranging from 0 to 5 were attributed according to the manufacturer interpretation scale and micro-haematuria was defined as urines coded ≥3.

Parasitological examination of urine and *S. haematobium* eggs count were performed after filtration of 10 ml of urine using Nytrel filters. The intensity of infection was categorized into two classes: light-intensity infection (egg count <50/10 ml) and heavy-intensity infection (egg count ≥50/10 ml). The geometric mean egg count of excreting individuals was used to assess the infection at community level.

*S. mansoni* infections were determined by preparing two 41.7 mg Kato-Katz thick smears from a single fresh stool specimen [17]. All stool specimens were also analysed for *Ascaris lumbricoides*, *Trichuris trichiura*, *Enterobius vermicularis*, *Taenia spp* and hookworm.

### Blood samples

A Hemocue photometer (HemoCue AB, Ängelholm, Sweden) was used to determine haemoglobin concentration from a capillary blood drop collected from the finger tip of each child, using sterile single-use material. Children were considered anaemic when haemoglobin was less than 11.5 g/dl [18].

A thick smear was made for the search of *Plasmodium falciparum*, but this examination was performed in the four villages of the region of Tahoua only. Children with asymptomatic malaria infection during dry season are supposed to have greater risk of chronic anaemia, knowing the great role played by malaria in the physiopathology of anaemia [13],[19].

### Ultrasonography

All the children underwent an ultrasound examination of the urinary tract to assess morbidity due to schistosomiasis using a portable ultrasonographer Aloka SSD 500 (Aloka, Tokyo, Japan) with a 3.5 MHz convex transductor. Bladder and urinary tract abnormalities were assessed and reported according to the WHO/Niamey protocol [20]. Bladder lesions were scored 0, 1 or 2, ureteral lesions were scored between 0, 3 or 4, and kidney distension was scored between 0, 6 or 8. By adding these elementary scores, an Echographic Severity Index (ESI) was calculated. In total, there were four classes of ESI: 1 = light impact, 2–4 = moderate impact, 5–9 = severe impact, ≥10 = very severe impact [16].

### Mass treatment

According to the national control programme procedures, praziquantel and albendazole were administered by a local team at the community level during the mass drug administration campaign, following the baseline data collection and 1 year later during the second mass drug administration campaign, following the control survey.

Praziquantel (using dose-pole corresponding to 40 mg/kg) and Albendazole (400 mg) were given to the target population regardless of infection status, during the mass drug administration campaign that took place 3–4 weeks after the surveys were conducted. Trained community drug distributors delivered the treatment in several distribution points in the villages. Schoolchildren were treated by teachers at school. Drugs were swallowed in presence of the drug distributor. Coverage rate reported by the national programme was approximately 68% for all the targeted regions (coverage rate was higher in school-based treatment than through community-based treatment).

### Data management and analysis

EpiInfo and SPSS software was used for data management and analysis. The Pearson's χ^2^, the Fisher's exact test, the Student's t test or the Kruskal-Wallis' test were used whenever appropriate. For paired analysis, the McNemar's χ^2^ was used. A *p* value <0.05 was considered significant. Logistic regression was used for multivariate analysis to identify the risk factors of anaemia.

## Results

### Baseline

A total of 1642 schoolchildren were enrolled in the study. The overall sex ratio (male to female) was 1.27: 1, but the sex distribution varied greatly according to the village, ranging from 14.9% to 73.3% for girls. The overall mean age was 8.7 years, without any significant difference between schools. The number in each age group was 532, 546 and 565 for the 7, 8 and 11 years old respectively.

The most commonly reported symptoms were bloody urine (41.8%), abdominal pain (28.5%) and pain when urinating (24.5%). A previous treatment against schistosomiasis was reported by 4.5% of the schoolchildren. No previous treatment had been administered at community level in any of the eight villages.

### Parasitological results

The overall prevalence of *S. haematobium* infection was 75.4% [95% Confidence Interval (CI): 73.2–77.5]. The prevalence varied from 43.6% to 97.7%, according to the village. Older children were more frequently infected than were younger ones (*p<0.01*). The overall prevalence of heavy-intensity infections was 21.8%. Intensity increased with increasing age: 16.4%, 21.8% and 27.0% in children aged 7, 8 and 11 years, respectively (*p<0.01*). Among the heavily infected children, 50 (13.9%) excreted more than 500 eggs/10 ml of urine. The geometric mean egg count among egg excreting children was 15.5 eggs /10 ml urine. Geometric mean egg counts were 11.7, 16.9 and 17.6 in children aged 7, 8, and 11 years respectively (*p<0.01*). The prevalence of infection was significantly higher in schools located in the south-western part of the country: 85.3% in the region of Tillabéry compared to 64.8% in the region of Tahoua *(p<0.01*). The prevalence of *S. haematobium* infection was significantly associated with reported haematuria and pain during urination (*p<0.01*).

*S. mansoni* infection was observed only in 2 schools: Sabon Birni (3%) and Sanguilé (1.1%).

Hookworm infection was observed in 3 schools; Sabon Birni, where the prevalence was 18.8 and in 2 other villages were the prevalence was 0.6%. Hookworm infection was not observed in the schoolchildren of the 5 other villages.

Very low prevalence (0.3 to 0.7%) of *Ascaris lumbricoides* infection was observed in 5 schools, while 3% of the schoolchildren were infected in 1 school (Sanguile) and no infection was observed in 2 schools (Kaou and Tabalak).

The overall prevalence of *P. falciparum* infection was 8% in the 636 tested schoolchildren, ranging from 3.8% to 15.8% according to the village (*p<0.03*). *P. falciparum* infection was neither associated with age, nor *S. haematobium* infection.

### Haematuria

The overall prevalence of observed gross haematuria was 6.9%, with a significant relationship with *S. haematobium* infection (*p<0.03*). Gross haematuria was observed in 21.4% of heavily infected children. The overall prevalence of micro-haematuria was 53.3%, differing significantly according to the village (*p<0.03*) and increasing significantly with increasing age (*p<0.03*). The prevalence of micro-haematuria was 9.6% in children not excreting eggs, 56.3% in children with light-intensity infections and 92.8% in children with heavy-intensity infections (*p<0.03*). Reported history of haematuria and pain during urination were significantly associated with the prevalence of micro- and gross haematuria (*p<0.03*).

### Haemoglobinemia

Overall, the mean haemoglobinemia was 11 g/dl (range: 5.3 g–17.3 g). The prevalence of anaemia was 61.6%, without significant difference between sexes. Haemoglobinemia was significantly related to age: 66.5% in children aged 7 years, 64.6% in children aged 8 years and 54.1% in those aged 11 years (*p<0.03*). Prevalence of anaemia was also statistically different according to the village (*p<0.03*). Anaemia was significantly associated with micro-haematuria (*p*<0.02), but not to gross haematuria. Overall, we found a significant association of anaemia with *S. haematobium* infection coded as a binary variable yes/no (*p* = 0.045), but not with infection coded into 3 levels of intensity. After adjusting for age, the association was present only in 8 year old children, where prevalence of anaemia increased significantly with increasing intensity of infection (*p<0.01*). The presence of anaemia was significantly associated with *P. falciparum* infection (*p<0.01*). Table 1 shows the prevalence of anaemia according to the presence, or absence, of several potential risk factors using bivariate analysis.

**Table 1:** Prevalence (%) of anaemia according to the absence or to the presence of several potential risk factors at the baseline data collection

Potential risk factor	n	Number of positive children	Prevalence of anaemia (%)		
			With risk factor absent	With risk factor present	*P*
*S. haematobium* egg excretion	1624	1230	57.4	63.0	*0.045*
Macrohaematuria	1624	111	61.3	66.7	*0.304*
Micro-haematuria	1614	861	58.6	64.3	*0.020*
Ultrasonographic abnormality of the urinary tract	1609	734	59.0	64.4	*0.025*
Bladder lesions	1608	667	59.9	63.6	*0.140*
Kidney distension	1595	66	60.4	81.8	*<0.001*
Ureteral distension	1614	66	61.3	66.7	*0.455*

### Ultrasound examination

The proportion of children presenting at least one ultrasound abnormality of the urinary tract was 45.8%, without significant differences between sexes or transmission patterns, and was significantly associated with age (37.8%, 46.0% and 53.0% in children aged 7, 8 and 11 years, respectively; *p<0.03*), to the intensity of *S. haematobium* infection (22.1% in children not excreting eggs, 47.5% in light-intensity infections and 68.1% in heavy-intensity infections; *p<0.03*) and to the presence of anaemia (*p* = 0.025).

Bladder wall abnormalities, evident in 41.6% of children, were the most frequently reported ultrasound abnormality. Their prevalence increased significantly with increasing age (*p<0.03*), with increasing intensity of infection (*p<0.03*), and was not associated with the presence of anaemia. Severe bladder wall abnormalities (either masses or pseudopolyps) were present in 15.5% of the children. Kidney and ureteral distensions were present in 4.2% and 4.1% of the children, respectively. Contrary to ureteral distension, kidney distension was significantly associated with the intensity of *S. haematobium* infection (*p<0.01*) and to the presence of anaemia (*p<0.03*).

The mean ESI, summarizing urinary tract morbidity due to *S. haematobium* infection, was 1.33 (range: 0–24) and the mean bladder score was 0.8 (range: 0–7). The mean ESI was not different between boys and girls. The mean ESI was significantly higher in pond-related transmission villages than in river-related transmission villages (*p<0.03*).

Multivariate analysis using logistic regression showed that the risk of anaemia was significantly associated with young age (OR = 1.7, 95% CI 1.3–2.2 for age = 7 and OR = 1.6, 95% CI 1.2–2.0 for age = 8), to the presence of ultrasound abnormalities of the kidneys (OR = 3.0, 95% CI 1.5–6.0) and to the village. When the same analysis was performed on the sub-sample of 4 villages where *P. falciparum* infection was documented, the predictors of anaemia were *P. falciparum* infection (OR = 2.5, 95% CI 1.3–4.7), ultrasound abnormalities of the kidneys (OR = 2.9, 95% CI 1.2–6.8) and the village (OR = 0.6, 95% CI 0.4–0.9 for Tabalak and OR = 0.4, 95% CI 0.3–0.7 for Kaou).

### Follow-up

A total of 89% of the initial sample group were re-examined one year after baseline data collection and the first round of treatment with praziquantel and albendazole. The mean age was 9.7 years and the sex ratio (male to female) was 1.27:1. A total of 482 eight year olds, 470 nine year olds and 484 twelve year olds were re-examined.

The overall prevalence of *S. haematobium* infection was 38% and 4.6% of children had heavy-intensity infections; only three (4.6%) among the latter excreted more than 500 eggs/10 ml. 56.2% of the initially infected children did not excrete eggs and 91.6% of heavy-intensity infections had cleared or become light intensity infection. However, the improvement of infection markers was different according to the village and prevalence was still above 60% in 3 schools (Kaou, Kokorou and Tabalak). In children from Tabalak, only minor changes in the prevalence and intensity of infection were observed (22.7% of heavy-intensity infections at baseline and 18.2% at follow-up).

Assessment of *P. falciparum* infection in the 4 schools tested in 2005 showed a significant increase of the prevalence from 8.6% to 17.1% (p<0.03). The rise of prevalence of malarial infection was observed in 4 schools, although only statistically significant in Rouafi (paired analysis, p<0.03).

The overall prevalence of all the morbidity markers of *S. haematobium* had decreased significantly at follow-up (Table 2). Among the 1412 schoolchildren which were re-examined, the overall prevalence of anaemia decreased from 61.9% to 50.4% (paired analysis, *p<0.03*) and the mean haemoglobinemia showed a significant increase of 0.39 g /dl to reach 11.4 g/dl (paired analysis, *p<0.01*). However, the change in prevalence of anaemia was not significant in children from Kaou, Kokorou, Mozague and Tabalak. At follow-up, anaemia was not associated with *S. haematobium* infection. Gross and micro-haematuria decreased from 7.1% to 0.4% (paired analysis, *p<0.03*) and from 53.5% to 6.0% (paired analysis, *p<0.03*), respectively. Prevalence of gross and micro-haematuria were each significantly associated with the intensity of *S. haematobium* infection (*p<0.03*). Anaemia was neither associated with micro-haematuria nor with gross haematuria. One year after treatment, the overall prevalence of ultrasound abnormalities of the urinary tract and the prevalence of ultrasound abnormalities of the bladder decreased from 45.6% to 15.2% (paired analysis, *p<0.03*) and from 41.6% to 14.7% (paired analysis, *p<0.03*), respectively. The mean global ESI showed a significant decrease of 1.04 (paired analysis, *p<0.03*).

**Table 2:** Evolution of the prevalence (%) of the main morbidity indicators between initial survey and re-assessment one year later (paired analysis)

	Comparison initial survey/one year post-treatment (paired analysis)					
Morbidity indicators	*Number of pairs*	No/No%	No/Yes%	Yes/No%	Yes/Yes%	*P* *
Reported haematuria †	1405	46.0	11.2	24.8	17.9	*<0.001*
Reported pain urinating †	1405	52.5	22.8	14.5	10.2	*<0.001*
*S. haematobium* infection †	1417	19.6	5.0	42.3	33.0	*<0.001*
Heavy-intensity infections ‡	1417	74.5	2.7	20.9	1.9	*<0.001*
Gross haematuria †	1417	92.6	0.2	7.1	0.1	*<0.001*
Micro-haematuria †	1405	45.2	1.4	48.8	4.7	*<0.001*
Anaemia †	1412	26.1	12.0	23.4	38.5	*<0.001*
Bladder lesion §	1407	52.8	5.6	32.5	9.1	*<0.001*
Ureteral dilatation †	1412	96.0	0.2	3.6	0.1	*<0.001*
Kidney dilatation †	1393	95.5	0.2	3.9	0.4	*<0.001*
ESI	1376	68.1	3.4	25.1	3.3	*<0.001*

*: McNemar's chi^2^ for paired samples

**†:** No = absent, Yes = present

**‡:** No = Not infected or light-intensity infection, Yes = heavy-intensity infection

**§:** No = no lesion, Yes = at least one lesion

**¶:** No = no impact or light impact, Yes = moderate to very severe impact

In 2006, the relationship between anaemia and *P. falciparum* infection was no longer observed.

Compared to those children who remained in the study cohort, the 216 children who dropped out after the initial survey differed significantly in the prevalence of *S. haematobium* infection (75.4% *vs.* 78%, respectively), but had less frequently heavy-intensity infections (22.8% *vs.* 16.5%, respectively). On the other hand, they did not differ in mean age (8.7 *vs.* 8.9 years, respectively), in the prevalence of anaemia (61.9% *vs.* 59.7%, respectively) nor in mean haemoglobinemia (11.04 g/dl *vs.* 11.03 g/dl, respectively).

## Discussion

According to the WHO guidelines [21], the aim of the Nigerien National Schistosomiasis Control Programme is to reduce schistosomiasis-associated morbidity by reducing the prevalence of heavy-intensity infections.

Our study, which compared baseline data collected before treatment and follow-up data collected one year post treatment, aimed to describe the evolution of *S. haematobium* infection and of anaemia prevalence in schoolchildren in 8 sentinel cohorts.

The predominance of boys in our samples reflects the overall low proportion of girls in full-time education in Niger. Only 11% of children of the initial sample did not take part in the follow-up survey. The characteristics of the children who dropped out of the study, for both ultrasound assessed morbidity and for anaemia, did not differ significantly from children who completed the follow-up. The children who dropped out also had slightly lower prevalence and intensity of infection at baseline.

### *S. haematobium* infection

Our data showed a high prevalence of urinary schistosomiasis at baseline, with a classical increase of both prevalence and intensity of infection with increasing age during childhood. Although studies have shown that both prevalence and intensity of infection is often higher among males in many surveys [22],[23], our data did not show such a relationship.

*S. haematobium* infection was well correlated to the prevalence of observed gross haematuria, confirming the value of this indicator for rapid assessment of infection at community level, as well as its intensity. Reported history of haematuria and pain during urination were both related to the infection, even if the schoolchildren underestimated it. These markers, have been used for rapid screening of urinary schistosomiasis [24].

According to the operating procedures of the national control programme, the praziquantel and albendazole treatment was administered by school teachers or health agents using the praziquantel “dose-pole” [25],[26] for praziquantel within 3–4 weeks following the baseline data collection (in November-December 2004 in Tillabéry region and in April-May 2005 in Tahoua region). Eleven to twelve months after treatment, the overall prevalence of *S. haematobium* infection had decreased significantly, from 75.4% to 38% and the prevalence of heavy-intensity infections had drastically reduced by 91.7%. These observations fully confirm the effectiveness of a single dose of praziquantel on infection, as demonstrated over the past two decades [27],[28].

However, in 3 schools, the prevalence of *S. haematobium* infection remained high, over 60%. In on village, Tabalak, the prevalence of heavy-intensity infections did not change at all. It is unlikely that this was solely due to re-infection, according to previous studies in Niger [16],[29] prevalence is unlikely to reach the initial level of intensity within a year of treatment. However, it has been demonstrated that *S. haematobium* reinfection patterns may vary strongly according to local epidemiological settings. Mass treatment took place after the high transmission period in the Tahoua region. In Tabalak where the transmission is year-round, the water level in local ponds usually decreases significantly during the hot and dry season. This reduction in water levels can induce a greater parasite concentration and thus a more severe infestation. Some alarming reports have been made since the 1990s about praziquantel treatment failures in *S. mansoni* infections [30], but despite large scale use of praziquantel in many countries there is currently no evidence to suggest that any *S. haematobium* has developed resistance to praziquantel as a result of its widespread use [31] and praziquantel can still be considered very effective. Furthermore, in the 4 villages where disappointing results were observed, the absence of mass treatment history should rule out a possible local problem of drug resistance induced by overuse or misuse of praziquantel. Although 75.1% of children in Tabalak reported having received tablets just after the baseline data collection, the absence of prevalence reduction is likely to be explained by the fact that praziquantel treatment was not administered as effectively as had been assumed. For children, confusion between albendazole tablets and praziquantel tablets could be quite easy and the drug delivery system in Tabalak will need to be assessed. In addition, transmission of *S. haematobium* in Tabalak will also be investigated.

### Soil-transmitted helminth infections

The very low prevalence of soil-transmitted helminth infection is in accordance with previous observations made in Niger [32] and in 2 other African countries, Mali and Chad [22],[33], areas that are subjected to climatic conditions comparable to the most inhabited regions of Niger. The single school where hookworm infection prevalence was over 1% was located in the Sudanian climatic zone, in the southern-most part of the country.

### Ultrasound-assessed morbidity

The nature and the frequency of ultrasound-assessed urinary tract abnormalities, observed in 46.2% of schoolchildren, with an association with both intensity of infection and with age are fully consistent with results from other studies [5],[34].

The effect of treatment on reducing the prevalence of both bladder and ureteral lesions was 78.2% and 96.3% respectively, a clear confirmation of the relevance of the control strategy. Previous surveys in Niger reported similar favourable results, showing the rapid regression of ultrasound-assessed bladder and ureteral lesions [16],[29],[35]. Many studies in other endemic areas also demonstrated the invaluable benefit of praziquantel administration on *S. haematobium* associated bladder morbidity [36].

At baseline, the prevalence of ultrasound assessed-kidney lesions was low (4.1%), compared to results from other studies which reported up to 43% of hydronephrosis in schoolchildren in hyperendemic village in the Niger River valley. Other results reported between 20.9% and 23.2% upper urinary tract lesions in two villages in an irrigated area of Niger [16],[29]. Contrary to other observations [37], in our study sample kidney dilatations were significantly associated with the intensity of *S. haematobium* infection. Although ultrasound-assessed kidney dilatations are not specific to urinary schistosomiasis, their regression following praziquantel treatment strengthens the likelihood of their relationship with infection. According to the literature, the observed clearance rate of kidney dilatations following praziquantel treatment may vary greatly, from low [38],[39] to very satisfactory [35],[40]. This may be explained by many factors, such as the length of the post-treatment follow-up or the age of lesions.

### Anaemia

Anaemia is considered a severe public health problem when its prevalence is over 40% within a population [41]. Anaemia is very common in developing countries and a multi-country survey in sub-Saharan Africa showed that it was generally a serious problem in schoolchildren [42]. Our results, showing a prevalence of 61.2%, confirm that anaemia in schoolchildren is a worrying public health problem in Niger.

Various causes can lead to anaemia including infections, low dietary iron intake, sickle-cell anaemia, malnutrition and inflammatory diseases. Although many cross-sectional studies and a small number of randomized controlled trials have addressed the relationship between schistosomiasis and anaemia, the conflicting results make the magnitude of the relationship unclear. Urinary schistosomiasis had been associated with anaemia in many studies. In a study in Niger, *S. haematobium* egg count and intensity of haematuria were negatively correlated to haemoglobin level, to haematocrit and to transferrin saturation, and positively associated with anaemia and iron deficiency [11]. Another Nigerien survey concluded that *S. haematobium* infections increased the risk of anaemia by 30% [10]. In Tanzania, haemoglobin concentration in infected children was 0.4 g/dl lower than in uninfected ones [43]. In Kenya, the intensity of *S. haematobium* infection was negatively correlated with haemoglobin level on a cross sectional basis [44]. On the other hand, a survey in Cameroon, in a context of concurrent malarial and schistosomal infections [15], did not establish a relationship between schistosomiasis haematobia and anaemia while anaemia correlated significantly with malaria infection. In Mali, in a survey focusing on interactions between helminth and malarial infections in children, results showed haemoglobin was similar in children infected with *S. haematobium* compared to those not infected [45]. Likewise, in a survey on anaemia in Tanzania, no relationship was observed with *S. haematobium*, whereas anaemia was significantly associated with hookworm infection [46].

Our results, showing a significant relationship between schistosomiasis haematobia and anaemia, were bordering on statistical significance in a bivariate analysis, and anaemia was not related to intensity of infection. In a previous analysis using an alternative haemoglobin threshold for anaemia (11 g/dl instead of 11.5, data not shown), no relationship was observed between infection and anaemia. Also, it is worthy to note that the prevalence and intensity of *S. haematobium* infection increased with increasing age, contrary to anaemia that was more prevalent in the youngest schoolchildren. The latter observation is consistent with the results found in Chad [22].

Our results showed that micro-haematuria increases with age as does the prevalence of schistosomiasis and that the prevalence of anaemia decreases with age. These findings raise the question about the significance of micro-haematuria in the prevalence of anaemia. By which mechanism does micro-haematuria contributes to anaemia? Why anaemia prevalence is higher in the age group where micro-haematuria is lower?

Lastly, in multivariate analysis, *S. haematobium* infection was not associated with anaemia. However, anaemia could be related to *S. haematobium* in an indirect way as it was significantly associated with micro-haematuria. The significant improvement of haemoglobin levels one year after praziquantel and albendazole treatment suggests that *S. haematobium* can contribute significantly to anaemia. In Burkina Faso where the SCI-supported schistosomiasis control programme provides also large-scale chemotherapy with praziquantel and albendazole, Koukounari and others found significant reduction in the prevalence of anaemia and a significant increase in haemoglobin concentration [47]. This confirms our findings on the benefit of praziquantel on anaemia.

Due to the study design, the causality cannot be formally established, but one can note that the 3 schools were anaemia did not improve corresponded to the 3 of 4 schools were *S. haematobium* infection showed only minor changes one year after praziquantel treatment. The overall very low prevalence of hookworm infection can rule out a significant contribution of hookworm in schoolchildren anaemia in Niger. Lastly, the observed improvement of anaemia is unlikely explained by the fact that some children dropped out of the survey before follow-up, as they did not differ significantly from the others, neither for *S. haematobium* infection, nor for anaemia prevalence.

The simultaneous delivery of several drug packages within the framework of the integrated neglected tropical disease control programmes (schistosomiasis, lymphatic filariasis, intestinal worms, trachoma, onchocerciasis) which are currently being implemented in several sub-Saharan African countries will probably involve an additional improvement of the prevalence of anaemia and the global health of the populations by the synergistic actions of the drugs on the worms, the ectoparasites and certain bacteria. Our study showed that anaemia can be a useful indicator for the monitoring of the impact of programs NTD as suggested by Bates et al. and Molyneux et al [48],[49].

Malarial infection is a well documented cause of anaemia in African children [13],[19]. The multivariate analysis performed on the sub-sample of 4 schools where data on *P. falciparum* infection were available showed that asymptomatic carriage of *P. falciparum*, kidney distension and the village were the only predictors of anaemia, while no relationship was found with *S. haematobium* infection. In Niger, 83.9% of children <5 years had an haemoglobinemia of <11 g/dl in a nationwide survey in 2006, with only minor differences between rural and urban settings [50], although both prevalence and intensity of *S. haematobium* infection are usually low in this age group. In total, *S. haematobium* alone cannot explain the high prevalence of anaemia in Nigerien schoolchildren. Actually, with 8.1% of children carrying *P. falciparum* at baseline, malarial infection could hardly explain the entire problem of anaemia in Nigerien children. Dietary iron availability should be investigated, as well as the prevalence of other known risk factors, such as sickle cell anaemia.

### Conclusions

Both *S. haematobium* and anaemia are highly prevalent in schoolchildren in Niger. Before treatment, anaemia was significantly associated with infection in a bivariate analysis, but this association was not observed in a multivariate analysis, while malarial infection was a significant predictor of anaemia. One year after systematic praziquantel treatment, we observed a significant decrease of the prevalence and intensity of infection and of morbidity. Though the prevalence of anaemia significantly reduced, prevalence was still 50.5%. The high prevalence of anaemia in Nigerien schoolchildren is likely a result of the combination of many risk factors and should be thoroughly investigated. The role of schistosomiasis on anaemia clearly deserves more studies. It would be interesting to study the interactions of malarial infection, schistosomiasis, soil-transmitted helminthiasis as well as nutritional status in the mechanisms of anaemia. Even though schistosomiasis represents only one of multiple causes of anaemia, the effect of praziquantel treatment on morbidity clearly indicates the benefits of schistosomiasis control programmes, which must be perpetuate.
